# The BioExperience Research and Entrepreneurship Challenge: An iGEM-inspired applied research program for BIOSTEM talent and skills development

**DOI:** 10.3389/fbioe.2022.1046723

**Published:** 2022-11-10

**Authors:** Hertek Gill, Mahdi Ahsan, Yara Khalil, Victoria Feng, Jessie Pearce, Tarasha Sharma, Mohamad Radwan, Austin Boucinha, Mads Kærn

**Affiliations:** ^1^ Ottawa Institute of Systems Biology, University of Ottawa, Ottawa, ON, Canada; ^2^ Department of Cellular and Molecular Medicine, Faculty of Medicine, University of Ottawa, Ottawa, ON, Canada; ^3^ Translational and Molecular Medicine Program, Faculty of Medicine, University of Ottawa, Ottawa, ON, Canada; ^4^ Biochemistry Program, Faculty of Science, University of Ottawa, Ottawa, ON, Canada; ^5^ Biomedical Science Program, Faculty of Science, University of Ottawa, Ottawa, ON, Canada; ^6^ Biopharmaceutical Sciences Program, Faculty of Science, University of Ottawa, Ottawa, ON, Canada; ^7^ Chemical Engineering Program, Faculty of Engineering, University of Ottawa, Ottawa, ON, Canada; ^8^ Canadian Synthetic Biology Educational Research Group (CSBERG), Centre for Applied Bioscience and Bioengineering Research (BioZone), University of Toronto, Toronto, ON, Canada; ^9^ Dufferin-Peel Catholic District School Board (DPCDSB), Mississauga, ON, Canada; ^10^ BioGroupe Canada Inc., Ottawa, ON, Canada

**Keywords:** experiential and work-integrated learning, engineered and synthetic biology, talent, skills development, undergraduate applied research

## Abstract

Post-secondary education is falling behind in delivering the talent and skills development needed to support the growth of biology-based economies and the demands of professional and research-based graduate degree programs. Here, we describe an applied research program, the BioExperience Research and Entrepreneurship Challenge, launched in May 2020 to mitigate the impact of the COVID-19 pandemic on undergraduate experiential learning programs at the University of Ottawa, Ontario, Canada. The program provided undergraduates with meaningful talent and skills development opportunities by implementing a student-centred, project-based learning approach inspired by the International Genetically Engineered Machine (iGEM) competition. We present evidence from participant exit surveys suggesting that the program delivers a high-quality learning environment and improves learning outcomes compared to traditional work-integrated learning. Notably, 84% of respondents reported an excellent or exceptional learning experience and significant or profound improvements in skills, such as leadership (72% of respondents), problem-solving (42% of respondents) and research preparedness (52%) that are difficult to develop in conventional academic programs. Remarkably, 60% of respondents report that the job-readiness training provided by the program is better or much better than traditional work-integrated learning. Our study demonstrates that a cost-effective and scalable alternative to the iGEM competition can improve talent and skills development in BIOSTEM fields.

## Introduction

The practical application of bioscience, biotechnology, bioengineering and biomedicine (BIOSTEM) research discoveries have had innumerable socio-economic benefits (Ganguly et al., 2014). This is evident by the rapid development of safe and effective RNA-based vaccines against the SARS-CoV2 virus (Dodd et al., 2021) and of CRISPR-based genetic engineering technology, which has the potential to cure serious human diseases (Jinek et al., 2012; Williams, 2014; Chan et al., 2021), facilitate equitable global economic development (Chui et al., 2020; Lange et al., 2021), reduce food insecurity (Shelton et al., 2020), replace unsustainable manufacturing and resource extraction practices (Kumar and Kumar, 2017; Degli Esposti et al., 2021), and mitigate the impact of environmental degradation and climate change (El Enshasy et al., 2020).

A highly educated workforce is essential to derive socio-economic benefits from technological advancements, and public post-secondary institutions have a social responsibility to ensure that this workforce can support economic development (Kirby, 2007). Unfortunately, post-secondary institutions are not adequately providing BIOSTEM graduates with the skills and competencies they need for successful careers in the bioeconomy (Wetzel et al., 2006; BioTalent Canada, 2021).

Several reports have documented deficiencies in post-secondary talent and skills over the past decade. A study from 2012 by the European Commission highlights insufficient skills development and a lack of industry collaboration as significant barriers to the European bioeconomy (New Skills for a European Bioeconomy-Conference Report, 2012). A 2013 survey of US college graduates revealed that while the majority believe they are well-prepared to enter the workforce, less than half of employers agreed (Mourshed et al., 2013). More recent studies by BioTalent Canada have revealed that many recent graduates lack skills and abilities that employers in the bioeconomy value the most, including non-technical essential skills such as problem-solving, critical thinking, communication, and collaboration skills (BioTalent Canada, 2021).

The expansion of work-integrated learning (WIL) has been proposed as a solution to address a growing labour shortage in the bioeconomy (Gamble et al., 2010). This type of learning can assist students by complementing their academic and technical skills with non-technical skills, such as collaboration, communication, and intrapersonal skills that are in high demand among employers but are challenging to develop in a classroom setting (Jackson, 2014; Edwards et al., 2015). WIL allows students to gain experiences relevant to their field of study through learning activities by incorporating experiences and practices from a professional setting (Billett, 2009; Sattler and Peters, 2013). For example, undergraduates pursuing research-oriented career paths often seek studentships in academic labs, while those pursuing careers in business seek corporate internships. Newer forms of WIL include community-based research and independent molecular biology (Cameron and Rexe, 2022), as well as a diverse range of extracurricular and community-organized competitions, hackathons, incubators, and accelerators, to meet increased demand for innovation and entrepreneurship skills development (de Villiers Scheepers et al., 2018).

Competition-based learning (CBL) is an appealing alternative to forms of traditional WIL, including studentships, internships, work-study and cooperative placements, because they deliver similar or enhanced learning opportunities to a higher number of learners with fewer human resources, particularly in project settings (Desai et al., 2014). The appeal of CBL arises because traditional WIL situates the learning in a highly structured environment under an authoritative figure responsible for defining what, when, and how tasks are to be completed. Because of this, the learner lacks the opportunity to set work objectives or participate in decision-making. In CBL, the learning is positioned in an environment created in collaboration with peers instead of a supervisor. This positioning shifts the learning responsibility to the students, who must develop strategies to acquire the knowledge and skills needed to compete. Accordingly, conventional WIL involves a structured work environment and continuous one-on-one engagement that are not required for CBL. Moreover, like other forms of student-centred project-based learning (Hoidn and Klemenčič, 2020), CBL creates opportunities for learners to engage in teamwork in ways that contribute directly to the development of intra- and interpersonal skills, such as communication, problem-solving, critical thinking, collaboration, and leadership skills (Abushakra et al., 2019).

The International Genetically Engineered Machine (iGEM) competition is a highly effective CBL program created almost 20 years ago to accelerate innovation in DNA-based biotechnology and bioengineering by applying engineering principles to molecular biology (Smolke, 2009; Vilanova and Porcar, 2014). The competition has facilitated the emergence of Synthetic/Engineering Biology as an applied science discipline and has contributed to numerous biotechnology start-up companies (Wright, 2020). It also helps students develop skills and abilities that bioeconomy employers have identified as lacking in recent graduates (Diep et al., 2021). However, the iGEM competition has a relatively narrow focus and must be complemented by talent and skills programs that can deliver similar learning opportunities more broadly across the bioeconomy.

The BioExperience Research and Entrepreneurship Challenge was created to replicate the exceptional learning outcomes of the iGEM experience and the entrepreneurial and opportunity-seeking mindsets of competition-based learning (Abushakra et al., 2019). The program was developed in 2020 at the University of Ottawa, Ontario, Canada, to mitigate the impact of public health restrictions imposed early in the COVID-19 pandemic. These restrictions caused a widespread loss of work-integrated learning opportunities that many undergraduate students rely on to earn a living while gaining hands-on experience over the summer. The program involves students working in teams to design and complete an applied research project defined by an industry or community partner that involves either a design project, consulting project, or research project.

More than 100 students have completed the program. Most used their participation in place of a cooperative learning work placement or a student research position and received a salary or a bursary. Others participated as unpaid volunteers. Participants working more than 10 h per week were included in most learning activities, and those receiving cooperative learning credits were graded based on the peer assessments. Students participating as research interns and volunteers did not earn academic credits for their work.

The analysis of program evaluations acquired through exit surveys shows that students had an overwhelmingly superb learning experience. In addition to developing technical knowledge, students report significant gains in skills and abilities associated with job readiness and research preparedness, including project planning and management, leadership, team management and collaboration, creative thinking, group thinking, adaptability, time management, organization, interpersonal relations, community engagement and entrepreneurship.

Our findings suggest that the BioExperience program offers a model for situated learning in post-secondary BIOSTEM education that requires relatively few resources. Although further research is necessary, evidence indicates that the program delivers high-quality talent and skills development in areas critical for biotechnology and biological engineering innovation and the growth of the bioeconomy.

## Materials and methods

### Program evaluation survey

Qualitative and quantitative data was collected through an online survey that participants were asked to complete immediately after the program ended. Google Forms hosted the online survey for the 2020 survey and SurveyMonkey for the 2021 and 2022 surveys. They consisted of questions with predefined Likert scale answers and questions with open-ended text answers. Likert Scale questions were chosen in consultation with members of the Faculty of Education at the University of Ottawa. Questions with open-ended answers asked participants to explain their ratings. The survey was mandatory for full-time participants and optional for part-time participants. Participants were predominantly University of Ottawa science and engineering undergraduate students. Other participants were University of Ottawa undergraduates in medicine and business, undergraduates from science, engineering, and business management at Carleton University and Western University, and secondary students.

### Data collection

Survey responses were exported and downloaded to the University of Ottawa IT network for filtering and analysis. There were complete responses from 59 unique respondents in 2020 (95% response rate), 34 respondents in 2021 (62% response rate) and nine respondents in 2022 (45% response rate). Participants who did not complete the survey were predominantly students who were volunteers with no formal association with the program. The anonymized but otherwise complete datasets are available upon request.

### Cohort compositions

Survey respondents were mainly science and engineering students (91%) in their third or fourth year. The most represented academic programs are biochemistry (17%), biotechnology (15%), biomedical mechanical or mechanical engineering (13%), biomedical science (11%), chemical engineering (9%) and software and computer engineering (9%). The percentage of female respondents is 59% (2020 cohort), 67% (2021 cohort), and 56% (2022 cohort). The rate of visible minority respondents is 56% (2020 cohort), 67% (2021 cohort), and 56% (2022 cohort).

### Data analysis

Participant responses to Likert scale questions were analyzed to determine the count and the percentages of responses for each option on the scale. The five Likert scale options were converted into numerical values from 0 to 4, with 0 corresponding to the most negative response option and four corresponding to the most positive response option. The resulting data were analyzed using R (version 4.3.1) and statistical functions included in the ggplot2 (version 3.3.3) and rstatixs (version 0.7.0) packages available from the Comprehensive R Archive Network. Scripts were written and executed using RStudio (Spotted Wakerobin release).

Qualitative analysis was conducted on participant responses to open-ended questions using an inductive approach.Table 1 is the codebook to guide the investigation. It developed and refined as suggested by MacQueen et al(2016) until the reliability of Fleiss’s kappa score ≥0.80 was achieved (McHugh, 2012). The refinement was conducted individually by five members of the analysis team (Vieira et al., 2010; Falotico and Quatto, 2015). Three randomly chosen research assistants were assigned participant response codes. Kappa values were calculated for each code for each question using formulas provided by Nichols et al(2011) and an overall kappa value for each question (De Vries et al., 2008). The final code(s) assigned to each response was restricted to code(s) appearing at least twice. A Python script was written to help determine if a code was present or not.

**TABLE 1:** Participant responses to open-ended questions were analyzed by associating each answer to one or more recurring themes assigned a unique code, synonym, and description. The recurrent themes are associated with the learning experience (EXP), the learning environment (ENV), and the learning outcomes (OUT).

	Theme code	Theme synonym	Theme description
ENV	COM	Communication	References to interactions among team members
	WFH	Location	References to the working from home
	COL	Collaboration	References to work within a team or other teams
	IND	Independence	Reference to student-led activities
	TIME	Organization	Reference to the timing of program components
EXP	GUI	Guidance	References mentorship, advice, feedback
	RWC	Relevance	Reference to practical or real-world application
	DIV	Opportunities	References to a variety of learning opportunities
	PCP	Impact	Reference to personal contributions
OUT	SOFT	Non-technical skills	References to “soft” skills development
	HARD	Technical skills	References to “hard” skills development
	DEV	Tangible outcomes	References to the work products and deliverables

### Study limitations

The design of the program evaluation survey imposes certain limitations. Notably, the quality of the responses could have been improved from the first iteration of the study by using more concise questions and detailed instructions. However, we decided against making changes to the initial survey questions to ensure consistency across the dataset. The study is also limited by insufficient research funding to organize and conduct the follow-up interviews and focus groups needed to confirm the thematic analysis results.

## Results

### Program development

The BioExperience program was launched to mitigate the impact of the COVID-19 pandemic on traditional undergraduate WIL programs at the University of Ottawa in Ontario, Canada. It was developed in April 2020 by faculty members from science, medicine, and engineering in collaboration with the University of Ottawa Co-operative (CO-OP) Programs Office and BioTalent Canada, a Canadian national non-profit human resources association for the biotechnology industry.

The rapid spread of the SARS-CoV-2 virus in early 2020 led to the closure of Canada’s international borders to most foreign nationals, the suspension of classes at Canadian post-secondary institutions, and the closure of all non-essential workplaces. Although the duration of these measures was unclear, they were expected to significantly impact undergraduate students’ ability to access the WIL programs that many rely on for meaningful employment and skills development during the summer term.

We designed the BioExperience program to replicate the student-centred approach of the iGEM competition by giving students a direct role in project design and team building. The freedom to self-manage is a central element of iGEM and is associated with essential intrapersonal and interpersonal skills (Farny, 2018). We also sought to design a program structure that addresses challenges experienced by students participating in the iGEM competition (Diep et al., 2021), including students choosing unrealistic and overly ambitious projects, participant dissatisfaction with mandatory tasks deemed irrelevant, and the breakdown of social cohesion within teams.

Students were given a direct role in project design by creating a context where a team of students is commissioned by a “client” to complete a research or entrepreneurship “challenge” on their behalf. The challenge is a short statement defining each team’s focus in terms of a problem, task, or question. Examples of Team Challenges are provided in Table 2. They must:

• Address an issue directly or indirectly related to an area of the bioeconomy,
• Require students to plan and engage in applied research while developing their skills and knowledge,
• Allow students to define their objectives and problem-solving strategies.

**TABLE 2:** Examples of Team Challenges from the 2020 BioExperience program. Full project descriptions and reports are available at https://biogroupe.ca/bioexperience/.

DIY 3D bioprinter systems for tissue engineering
Tissue engineering, which seeks to regenerate the tissues and organs in our bodies from the combination of cells, scaffolds, and bioactive signals, has shown promise for the treatment of injuries and diseases and for the development of improved *in vitro* models to study physiological and pathological cellular processes. This project aims to generate comprehensive knowledge of these technologies and develop a detailed plan to build a novel, cost-effective, and versatile 3D bioprinting system that can generate complex multicellular and anisotropic tissue structures for various applications
**Experimental Strategies for the Craft Beer and Vodka Community**
To meet the increasing demand for highly skilled workers in the microbrewery and craft distillery, the Faculty of Engineering is developing a pilot-scale microbrewery and craft distillery. Once established, this facility will provide an experiential learning environment where students can acquire knowledge and hands-on expertise to increase their job readiness and meet the recruiting needs of a rapidly growing sector of the bioeconomy. To better prepare students for employment in the microbrewery and craft distillery sector, the proposed project will investigate economic small-scale production alternatives for local craft businesses and develop experiential learning activities and experimental strategies for small-scale brewing and distillation systems
**Biodigital Convergence—Strategic Foresight in an Era of Disruption**
Advanced DNA-based biotechnology is radically changing our economy, ecosystems, and society. Biodigital Convergence—the merging of digital and biological technologies and systems, has the potential to change the way we work, live, and play. This project aims to examine how future biodigital technologies could be woven into our lives and potentially transform our understanding of ourselves and the natural world, the meaning of human connection and the essence of humanity itself
**Emerging Biotechnologies for COVID-19 Point-of-care Testing**
Diagnostic testing of viral infection often involves processes prone to error and require specialized equipment. Several biotechnologies have recently been developed to address these issues and enable cost-effective and reliable point-of-care testing. This project aims to generate comprehensive knowledge of the biological principles behind SARS-CoV-2 diagnostic testing methods and to develop a design implementation of rapid COVID-19 point-of-care testing

It is helpful to think of a challenge as a task outsourced to a student team, much like an organization might outsource work to a consulting company. In both scenarios, the “client” defines general expectations and parameters and delegates the details to “consultants.” This approach transfers the project’s ownership and responsibility to the students, who must collaborate to identify project objectives and develop the appropriate research plans and problem-solving strategies. It also gives teams the flexibility to ensure that their project accommodates the interests, skills and learning needs of all team members. We envisioned adopting this approach would translate into an engaging and motivating learning experience by allowing participants to focus on activities that align with their interests and long-term aspirations.

We initially focused on projects that students could complete without access to a laboratory or other physical workspaces, including design projects, consulting projects, business development projects and projects involving literature reviews. We did this in anticipation that students would not be able to return to in-person learning. However, challenges requiring field- or laboratory work or other in-person activities are allowed if the team client or academic advisor provides the required facilities, resources, and training.

We also focused on projects endorsed by a faculty member committed to providing guidance and project management support. We did this to ensure that teams could set achievable goals, develop realistic research plans, and maintain a safe and productive learning environment. Projects can be initiated by a non-academic client, including, for example, private companies, business development organizations, and government agencies, or by faculty members. In the latter case, the faculty member acts as the team client and academic advisor.

We implemented a stacked recruitment process that gives students a key role in team building and project development. First, an initial cohort of 10 “Team Leads” was recruited to identify the skills and abilities needed to complete their challenges. Students in the second cohort were recruited to build a core team, while students in the third cohort were recruited to fill any remaining gaps. The idea was for faculty advisors to interview and assign Team Leads to specific projects and for Team Lead to work with the advisor to identify recruitment needs, write job postings, and conduct applicant interviews with support from their advisors. We also anticipated that this would help teams develop and maintain social cohesion by clarifying each team member’s purpose and roles, and responsibilities.

The launch of the program was made possible by a commitment in mid-April 2020 by BioTalent Canada to pre-approve 50 University Ottawa CO-OP students for up to CAD 7,500 for their Student Work Placement Program (SWPP). Shortly after, the University of Ottawa agreed to provide administrative support and complement the BioTalent Canada funding with CAD 4,500 per student through the University of Ottawa Work-Study program.

A job posting advertising the 10 Cohort I Team Lead positions was released on 29 April 2020. It emphasized that ideal candidates should be able to work harmoniously with others in a team-based and project-oriented environment, be self-directed and self-motivated, comfortable with initiative and leadership, and have an interest in project development and management. Among 56 applicants, 11 Team Leads (one more than initially expected) were recruited to develop the applied research projects. Subsequent Cohort II and Cohort III recruitment, which was extended to accommodate students who became unemployed in mid-June, resulted in the enrolment of 59 students. Remarkably, all students completed the program, and only one project failed to proceed beyond the initial development phase. This failure was caused by insufficient support from a faculty member. The orphaned student was successfully integrated into another project where they became responsible for a sub-project.

### Core components

**FIGURE 1:**
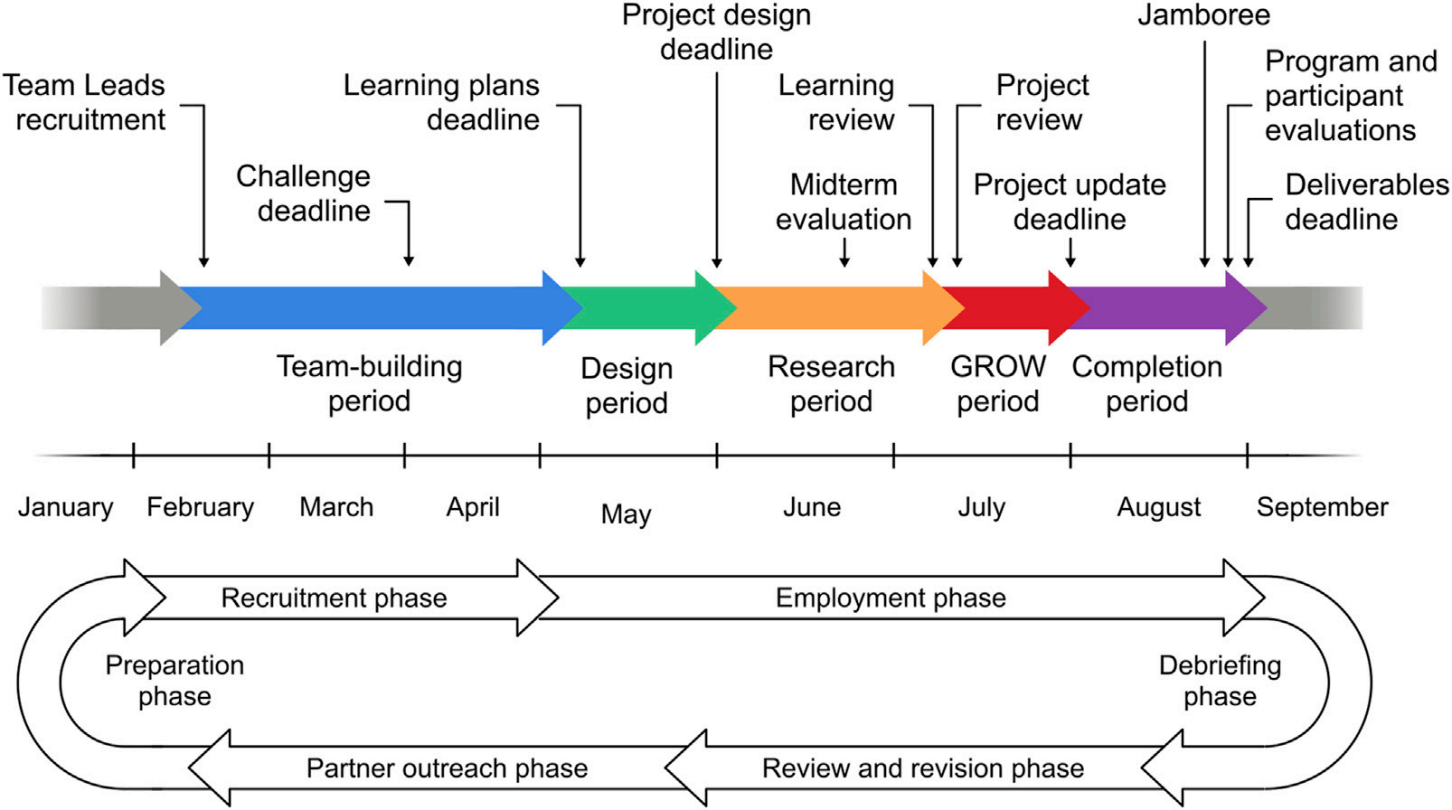
Program overview. Schematic illustration of milestones and timelines spanning a recruitment period from January to the end of April and an employment or work period from May to August. The recruitment phase and the first week of the work period are dedicated to team building. The remaining employment period consists of a design period, a research review, a review/GROW period, and a completion period. The program ends with a debriefing and a review and revision period. The planning for the following summer starts with a partner outreach period and is followed by a recruitment preparation period.

The BioExperience program has evolved since its first iteration but has retained a structure composed of five core components, including team building, project design, research, review, and completion. The order of the components and their associated milestones are depicted in Figure 1. They are envisioned to span 8 months, with a low-intensity recruitment phase from January to April and a high-intensity period from May to August. The figure also depicts program phases that do not involve students directly. They include a debriefing phase involving discussions with participating faculty members and non-academic partners, and a program review and revision phase to identify areas where the program can be improved. The solicitation of challenges for the next summer starts in November faculty members and non-academic partners, and preparations for the recruitment of Team Leads start in December.

#### Team-building period

The team building period begins with recruiting one or more Team Leads and ends with a learning activity where students share personal learning plans with their teammates. As mentioned in the previous section, Team Leads, or Cohort I students, are recruited to work with the Team Client to develop a project outline that can guide the team-building process. The project outline helps the Team Leads create job postings and conduct student interviews by identifying the skills and competencies needed to complete the project.

This team-building process allows Team Leads to practice their leadership and human resources skills. A faculty member supports Team Leads during the hiring process to ensure that program expectations and the roles and responsibilities of Cohort II and Cohort III recruits are communicated accurately. Notably, it is essential to firmly establish that the responsibility of the Team Leads is to facilitate the development of a supportive and inclusive learning environment where students engage in work that aligns with their interests, skills, and learning needs. They are not responsible for the project’s overall success and are not permitted to assume the role of an employer. In other words, they cannot instruct or supervise the work of other team members.

The first week of the employment period focuses on developing personal learning plans. The goal is to encourage students to take ownership of their learning and facilitate constructing a learning environment that accommodates all team members’ interests, goals, and needs. The personal learning plan is a mandatory component of BioTalent Canada’s SWPP program and consists of answers to four open-ended questions:

1. Where am I now, and where do I want to go?
2. Which skills and competencies do I need to succeed?
3. What learning activities will help me acquire these skills?
4. How can my teammates and advisors support me?

Students participating in the program’s first iteration found these questions challenging, and many could not articulate clear goals or identify the skills and abilities they would need to achieve them. Accordingly, a team-based learning activity called “Own Your Learning” was created to get students PRIMED for self-directed learning through self-reflection and group discussions with team peers. Preparing students PRIMED for learning involves:

• Making it more apparent to students what they want to accomplish and why (Purpose),
• Letting students take charge and ownership of their learning (Responsibility),
• Encouraging students to be their own and each other’s teachers (Independence),
• Creating a sense of belonging, meaning and accomplishment (Motivation),
• Facilitating personal growth and professional development (Evolution), and
• Helping students stay focused as they progress through the program (Direction).

The question “where do I want to go?” was challenging for many students. Accordingly, in 2021, students were asked to create a list of specific, measurable, attainable, relevant, and timely (SMART) learning goals (O’Neill and Conzemius, 2006) before completing their learning plan. Students were specifically asked to answer the following questions:

• What will achieving your learning goals allow you to do? Why is this ability important to you (Specific)
• How will you know that you have successfully reached your goals? What are ways to assess your progress (Measurable)
• Are your goals realistic? What are the skills and competencies you need to achieve them? Do you already have the necessary expertise? Are resources available to access or acquire them (Attainable)
• How are your goals related to your personal and professional aspirations? Do they make sense in the context of the BioExperience program and your Team Challenge (Relevant)
• Is this the right time to pursue these specific goals? Why is it important to attain them now rather than later? Could your time be spent on something more substantial (Timely)

Clarifying the purpose of the personal learning plan using the PRIMED concept and introducing SMART learning goals was further augmented with surveys to help students identify and prioritize specific skills and abilities and to monitor their progress throughout the program.

#### Design period

The project design phase occurs after developing personal learning plans to ensure that students have an opportunity to create a project that supports the learning of all team members. During the design phase, teams are expected to use the project outline created by the Team Lead as the foundation of a detailed account describing how they plan to complete their challenge.

Students are asked to answer three questions individually:

1. Why is addressing this challenge significant?
2. Who will benefit from the work done by the Team?
3. How will they benefit?

The goal is to help the student connect to the project on a personal level before working with their peers to create a shared vision of what they would like to achieve as a team. The shared vision is expected to support collaboration and decision-making and strengthen social cohesion within the Team. It also helps teams adopt a backward design strategy (Emory, 2014) that focuses on the project’s overall purpose and encourages students to think creatively, take risks and explore opportunities as they emerge.

The development of a purpose-driven project is facilitated by teams answering a series of sequential questions in a project design guide. The questions, in order, are:

1. What do you aspire to accomplish by completing your project (Project Goal)
2. Why is reaching the goal important? Who will benefit? How (Project Purpose)
3. How will you know that you are successful? What will you create to demonstrate to your client that you have achieved your goal (Tangible Outcomes)
4. What are the significant steps to be completed before you can generate these outcomes (Specific Aims).

To further support research planning, teams are asked to produce a step-by-step research plan describing what they intend to do to complete each specific aim, including a Gantt chart (Geraldi and Lechter, 2012) to visualize timelines. They are also asked to describe the knowledge and skills the Team needs to acquire, how they will be developed, what risks might prevent the Team from succeeding, and how it will mitigate them.

#### Research period

The research period is when teams execute their research plan. Teams are expected to meet with their faculty advisors at least once per week and to provide brief progress updates at a weekly meeting of all participants. This meeting aims to connect students from different teams and create team collaboration opportunities.

Students must work closely with their teammates and advisors. They are expected to have a daily team meeting to coordinate their work and share their findings, support the learning of others, and seek assistance from teammates when needed. They are also told that they are expected to collaborate to define, coordinate, and delegate tasks consistent with individual team members’ interests and learning goals. This expectation creates rich opportunities for students to engage in negotiation, mediation, and conflict resolution.

Team leads are tasked with maintaining a positive and productive learning environment and are supported in this responsibility through weekly meetings with the program director and other team leads. The program director is required to mediate conflict, resolve differences of opinion, or restore social cohesion. However, it is crucial to convey to all participants that the program facilitates professional development and that challenges are opportunities to improve interpersonal and intrapersonal skills.

The midpoint of the research period includes a peer- and self-assessment survey. The self-assessment asks students to reflect on their learning progress by rating their proficiency in the skills and abilities they were asked to prioritize earlier in the program. They are also invited to identify up to three skills and abilities they feel they have developed and describe how they demonstrated high proficiency. The peer-assessment questionnaire is identical to the self-assessment questionnaire and asks students to rate their teammates’ proficiencies the same way they rated themselves. They are also invited to identify up to three skills and abilities that stand out and to describe how the teammate demonstrated proficiency. The peer assessments provide valuable feedback and give participants a complete picture of their learning journey by identifying areas where progress has been made and areas to develop further.

#### Review (GROW) period

The research period ends with a review period where teams assess their progress and reassess their project design. The review was done using a GROW project planning model (Kang et al., 2021), which asks teams to answer the following questions:

• What is it that you are trying to achieve (Goals)?
• What progress have you made? What is impeding your progress (Reality)?
• What is the ideal solution? What are realistic solutions (Options)?
• What will you do now? How will you do it (Will)?

Completing the GROW activity helps teams ensure that their goals are still achievable in the remaining time based on what they have learned during the research period and gain an increased understanding of the time it takes to complete various tasks. It also allows teams to revise their tangible outcomes in case the initial plan was overly ambitious or unexpected complications emerged during the research period.

#### Completion period

The final period of the program begins after the review period. Teams have now assessed their plans and made the necessary changes to ensure their objectives are achievable in the time remaining in the program term. This period is termed the “Go Period,” where teams focus on completing their tangible outcomes.

The Completion Phase culminates in a final celebratory Jamboree when each Team presents their achievements. This presentation acts as an opportunity for teams to showcase their work, celebrate each other’s success, and give members of the public a window into the work achieved by the teams. The presentations are not evaluated as the focus is on understanding how obstacles were overcome and what future steps for the projects might be.

### Auxiliary components

In 2021, we began offering weekly instructor-led learning activities covering various topics, from researching literature using databases to writing a personal learning plan and creating compelling pitch presentations. We did this because it was apparent that many students could not reach program milestones independently. We also worked with BioTalent Canada to provide participants with access to their “Skills for Success” online workshop series. These modules cover essential interpersonal skills such as communication, collaboration and problem-solving as well as technical skills such as quality assurance/quality control and good lab practices. This auxiliary component is not easily reproduced.

### Skills improvements

The BioExperience program aims to help participants improve skills essential for employment in the bioeconomy and graduate studies in a BIOSTEM field. To assess if this objective was achieved, we examined quantitative Likert-scale ratings by the participants of their advancement in eight higher-order non-technical skills, including self-management, problem-solving, critical thinking, research, collaboration, teamwork, communication, and leadership. Participants were asked to rate the degree to which the program helped them improve relevant proficiency on a five-point scale from none to profound.

To assess participant skills and competency development, we calculated the percentage of respondents reporting high improvement in their ability to complete specific tasks and activities. Table 3 provides an example for the rating of improvement of leadership skills. This skill is associated with seven sets of abilities, such as setting team goals and resolving conflicts among team members. In the example, 74% of respondents rate their ability to “build and maintain team cohesion, morale and discipline” improved significantly or profoundly. In comparison, 51% give this high rating for their ability to “resolve conflicts and negotiate differences of opinion among team members”.

**TABLE 3:** Leadership skills development. Participants were asked to rate the improvement in their ability to complete tasks associated with leadership. The rating options were none, minor, moderate, significant, and profound. **(A)** Fraction of respondents who rated their improvement in specific abilities as significant or profound. **(B)** Fraction of respondents who rated their overall skill improvement as high (significant or profound) or low (none or minor). The overall rating *r* of each respondent was determined by assigning to each rating option a numeric value from zero (none) to four (profound) and averaging these values. The number of participants providing a high and low overall rating is the number of partitions with *r* ≥ 2.5 and *r* < 1.5, respectively.

(A) Description	N	Count	Percent (%)
Identify and set realistic project goals, tasks, and priorities	89	63	71
Manage, delegate and coordinate project tasks to match the interests and competencies of team members	89	65	73
Recognize the capabilities of individual team members through inclusion in decision-making	89	65	73
Build and maintain team cohesion, morale, and discipline	89	66	74
Provide support and guidance to team members	89	66	74
Ensure that team members can contribute in ways which support their learning	89	60	67
Resolve conflicts and negotiate differences of opinion among team members	88	45	51
**(B) Overall rating fractions**			
High overall rating (significant or profound improvement)	89	64	72
Low overall rating (none or minor improvement)	89	5	6

To get a sense of the overall rating of individual respondents, we also calculated an aggregate rating to better assess the average improvement rating across the sets of abilities used to assess higher-order skills. In this calculation, we used a linear numerical scale from zero (no improvement) to four (profound improvement) and calculated an overall rating score *r* for respondents by averaging. The fraction of high and low ratings was then computed by counting the respondents with an overall rating of *r* ≥ 2.5 or *r* < 1.5, respectively. In the example in Table 3, 64 respondents (72%) rated their improvement in leadership skills as high (i.e., significant, or profound improvement). In comparison, five respondents (6%) rated their improvement as low (i.e., no, or minor improvement). The remaining respondents rated their improvement as moderate.

**FIGURE 2:**
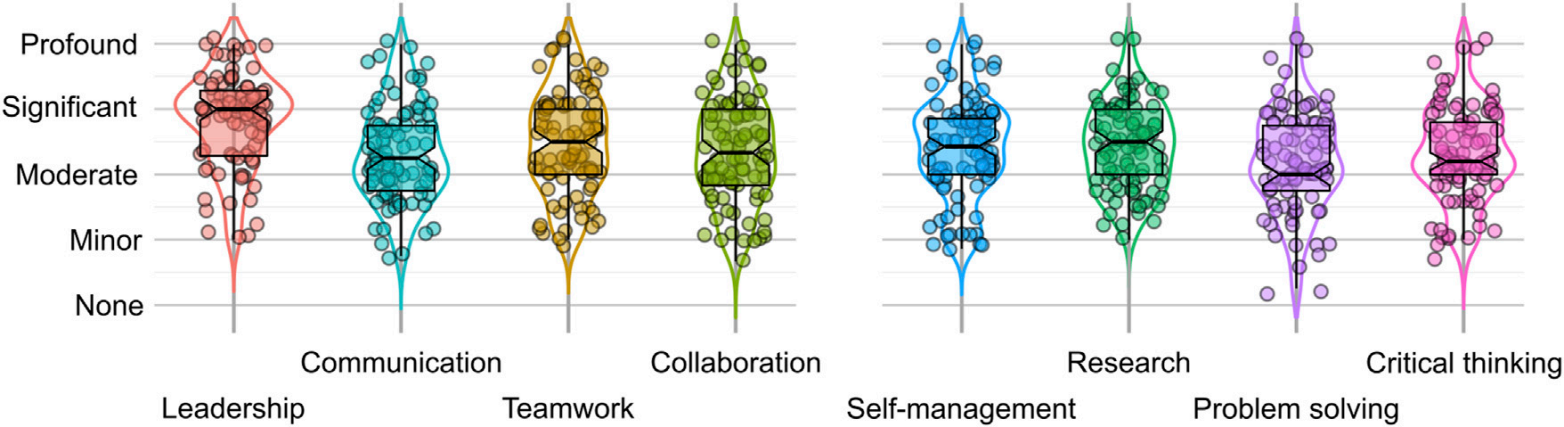
Higher-order skills improvement. Participant response distributions for higher-order skill groups. Data points indicate the average respondent rating for each group (see Table 1 for details). Curves represent the distribution of data points (violin plots), and the notched boxes represent the 2nd and 3rd quartiles of the data (box plots).

The ratings for seven higher-orders skill groups suggest that most participants saw skill improvements. Figure 2 depicts the distribution of all respondent rating scores for each skill group on a continuous scale from zero (no improvement) to four (profound improvement). The median rating scores lie between 2.0 (moderate) for problem-solving and 3.0 (significant) for leadership. While individual scores vary considerably within each group, the box- and violin plots used to represent the data clearly illustrate that most respondents reported a moderate improvement or higher. The lowest first quartile value is 1.75 for communication and problem-solving, meaning that at least 75% of respondents saw medium (1.5 ≤ *r* < 2.5) or high (*r* ≥ 2.5) overall improvement.

The percentage of respondents with high (*r* ≥ 2.5) and low (*r* < 1.5) overall improvement ratings provide insight into the ability of the program to facilitate the development of higher-order skills and competencies. Table 4 summarizes the results of our analysis by presenting the fraction of responses indicating high and low overall improvement rates. The fraction of respondents rating their improvement as high is around 50% for most skills, with significant or profound improvement, most common in leadership skills (74%) and research skills (60%). The table also includes the survey questions with the highest and the lowest number of responses with an improvement rating of significant or profound. For example, in the research skills, the ability to “develop and manage research or product development projects” was the ability improved by the most participants (54%), while the ability to “conduct and organize literature or technical reviews'' was the ability improved by the fewest participants (47%).

**TABLE 4:** High-order skills development. The percentage of respondents rating their improvement as high (significant or profound) or low (none or minor). The most (least) frequently improved abilities are the abilities that most (least) respondents identified as significantly or profoundly improved.

Skill	High rating (%)	Low rating (%)	Most frequently improved	Least frequently improved
Self-management	49	16	Be reliable and consistent in the completion of tasks; Set and manage personal schedules and priorities (57%)	Manage mental and physical health (30%)
Problem solving	42	14	Integrate multiple perspectives and disciplines in problem-solving strategies (51%)	Perform data analysis and hypothesis generation (28%)
Critical thinking	40	12	Integration of conflicting evidence or viewpoints (51%)	Be aware of and able to challenge biases, inferences, and assumptions (38%)
Research	52	6	Develop and manage research or product development projects (54%)	Conduct and organize literature or technical reviews (47%)
Collaboration	48	14	Negotiate and distribute tasks fairly (54%)	Resolve conflicts with or among others (35%)
Teamwork	52	11	Share knowledge and support the learning of others (58%)	Reach consensus through negotiation and compromise (40%)
Communication	44	8	Use plain language to communicate complex information (58%) clearly	Develop clear technical standards, procedures, protocols, or guidelines (38%)
Leadership	72	6	Build and maintain team cohesion, morale, and discipline; Provide support and guidance to individual team members (74%)	Resolve conflicts and negotiate differences of opinion (51%)

### Personal development and achievements

**FIGURE 3:**
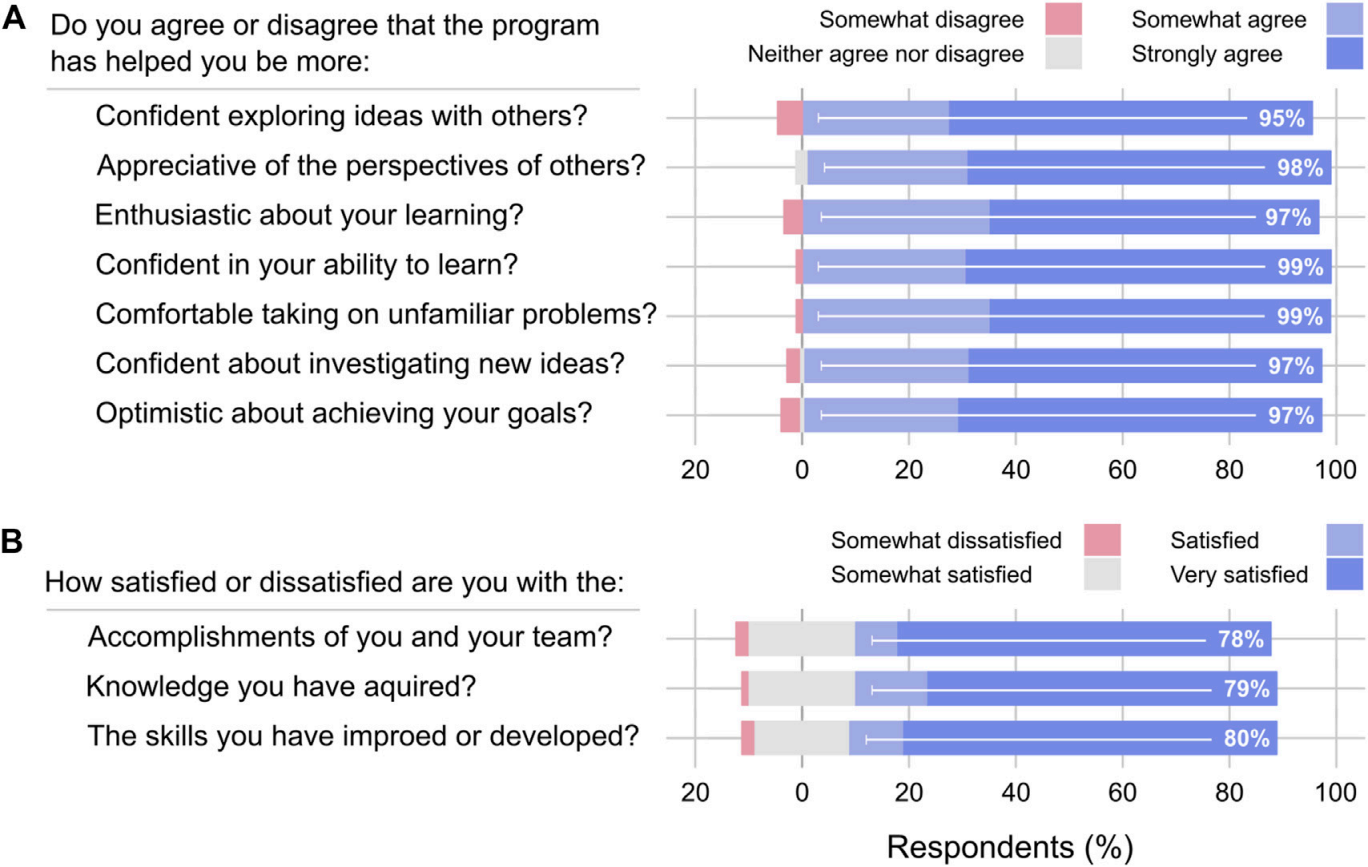
Learning outcomes. **(A)** Personal development. **(B)** Personal achievements. Each horizontal bar includes the percentage of respondents selecting the two highest options on a five-point Likert scale. None of the respondents chose the lowest option (unacceptable, strongly disagree, or very dissatisfied).

The personal development of participants was assessed through quantitative and qualitative means. In the quantitative approach, we were particularly interested in gauging to what degree participants felt the program had helped them improve in areas important for self-motivation and self-direction and developing an inquisitive mindset. The results are summarized in Figure 3A.

Almost all respondents agree that the program helped their personal development in one area or another. For example, 95% of respondents agree or strongly agree that the program had made them more “confident about my ability to learn,” 99% agree or strongly agree they are more “comfortable taking on unfamiliar problems,” and 97% agree or strongly agree that they are more “positive about achieving my goals.” To further assess personal development, we asked participants to describe up to three things they learned that would be valuable in their future (data not shown). The most frequently referenced themes were non-technical skills (75% of participants) and technical skills (65%). Recurring subjects were leadership, communication, teamwork, adaptability, self-management, entrepreneurship, project planning, programming, engineering design, and biotechnology and bioeconomy knowledge.

We also asked participants to rate their satisfaction with their achievements and to describe what they were most proud of having accomplished in the program. The results are summarized in Figure 3B. Almost all respondents reported that they were somewhat satisfied, satisfied or very satisfied with their accomplishments (98%), the knowledge they acquired (99%) and the skills they developed (98%).

Remarkably, roughly two of every three respondents expressed that they were very satisfied with their achievements in at least one area. In open-ended answers, 61% of respondents referenced a work product or project deliverable as their proudest accomplishment. They also identified non-technical skills (20%) and technical skills (17%), with communication, leadership, and collaboration often recurring in responses referencing non-technical skills. Software and web development, product development, and research abilities were the most frequently recurring technical skills.

### Program quality

**FIGURE 4:**
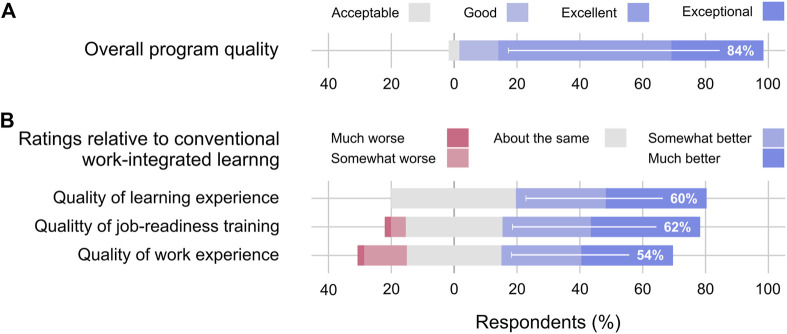
Program quality. **(A)** Distribution of responses to the survey question “How would you rate your overall experience?“ **(B)** Distribution of ratings comparing the BioExperience to conventional WIL programs. The quality of the work experience refers to the meaningfulness of the work as perceived by respondents. Horizontal bars include the percentage of respondents selecting “Excellent” or “Exceptional” in **(A)** and “Somewhat better” or “Much better” in **(B)**.

We asked participants to provide an overall rating of the program and to describe what worked well for them in the program. Remarkably, 84% of respondents rated the overall experience as “excellent” (49 respondents) or “exceptional” (26 respondents). The remaining respondents gave a rating of “good” (11 respondents) or “acceptable” (3 respondents). This data is presented in Figure 4A. In the answers to the open-ended question, the three most frequently identified themes were communication (43% of respondents), collaboration (40%) and technical skills development (21%). Recurring sub-themes were team meetings and software for communication, delegating tasks, setting common goals, peer support for collaboration, and writing and software use for technical skills.

We also asked participants to describe why they might complete the program in the future or recommend it to a peer. Respondents frequently referenced the ability to develop technical skills (24%), non-technical skills (20%), and learning opportunities (24%). Subjects mentioned frequently by respondents included leadership opportunities, the development of self-awareness, the management of research projects, and the ability to choose projects related to their academic studies and career goals. The most frequent reasons why respondents would recommend the program are the learning opportunities (35% of respondents) and the development of non-technical (29%) or technical skills (21%). Other common reasons participants recommend the program include the student-led learning environment (19%) and collaboration (11%). Recurring subjects were related to the participant’s ability to learn about things they are interested in or passionate about in a diverse and inclusive environment.

### Comparison to conventional WIL

We asked participants who had previously participated in WIL to provide a comparative rating regarding the quality of the learning experience, the value of the program for job-readiness training, and the ability to do meaningful work. The summary results are presented in Figure 4B. All respondents rated the quality of the learning experience as similar or better than a previous conventional WIL experience, and more than half rated it somewhat better or much better. Similar ratings were obtained for job-readiness training and work experience, where 62% and 54% rated the quality of the BioExperience program better than conventional WIL.

We also asked participants to explain their ratings. In the context of the learning experience, the most frequent themes were technical skills development (44% of respondents), non-technical skills development (32%), collaboration (27%) and independence (24%). In the technical skills area, recurring subjects were increased knowledge of the bioeconomy and biotechnology research, the development of entrepreneurial skills, and proficiency in research design and management. In the non-technical skills area, respondents frequently mentioned project development and management, teamwork, and self-directed learning, while working with like-minded students, problem-solving and self-direction were common subjects in the collaboration and independence themes.

In terms of improved job-readiness training and work relevance, respondents frequently referenced the relevance of their work to real-world problems (26% of respondents) and the development of non-technical skills (18%) and technical skills (16%). Recurrent subjects include helping others and addressing societal challenges, teamwork, organization, self-motivation, communication, and leadership in the non-technical skills theme, research skills, technical knowledge, entrepreneurship, engineering design and scientific writing in the technical skills theme.

### Areas for improvement

To identify areas of improvement, we asked students to describe something that did not work well for them, one thing they could change about their work-related interactions, and one thing they would add to the program if they could. The responses to the two first questions were consistent, with the most frequently referenced identified being communication, workplace location (i.e., working from home), and guidance. Guidance and learning opportunities were the most identified areas of improvement. However, there was a significant difference between the 2020 and 2021 program iterations. While 38 responses from the 2020 survey referenced guidance or opportunities as areas of improvement, corresponding to 45% and 36% of respondents, they were only referenced in one response from the 2021 survey. In the 2021 survey, the most frequently referenced areas of improvement were non-technical skills (38%), technical skills (37%) and program structure (38%).

**FIGURE 5:**
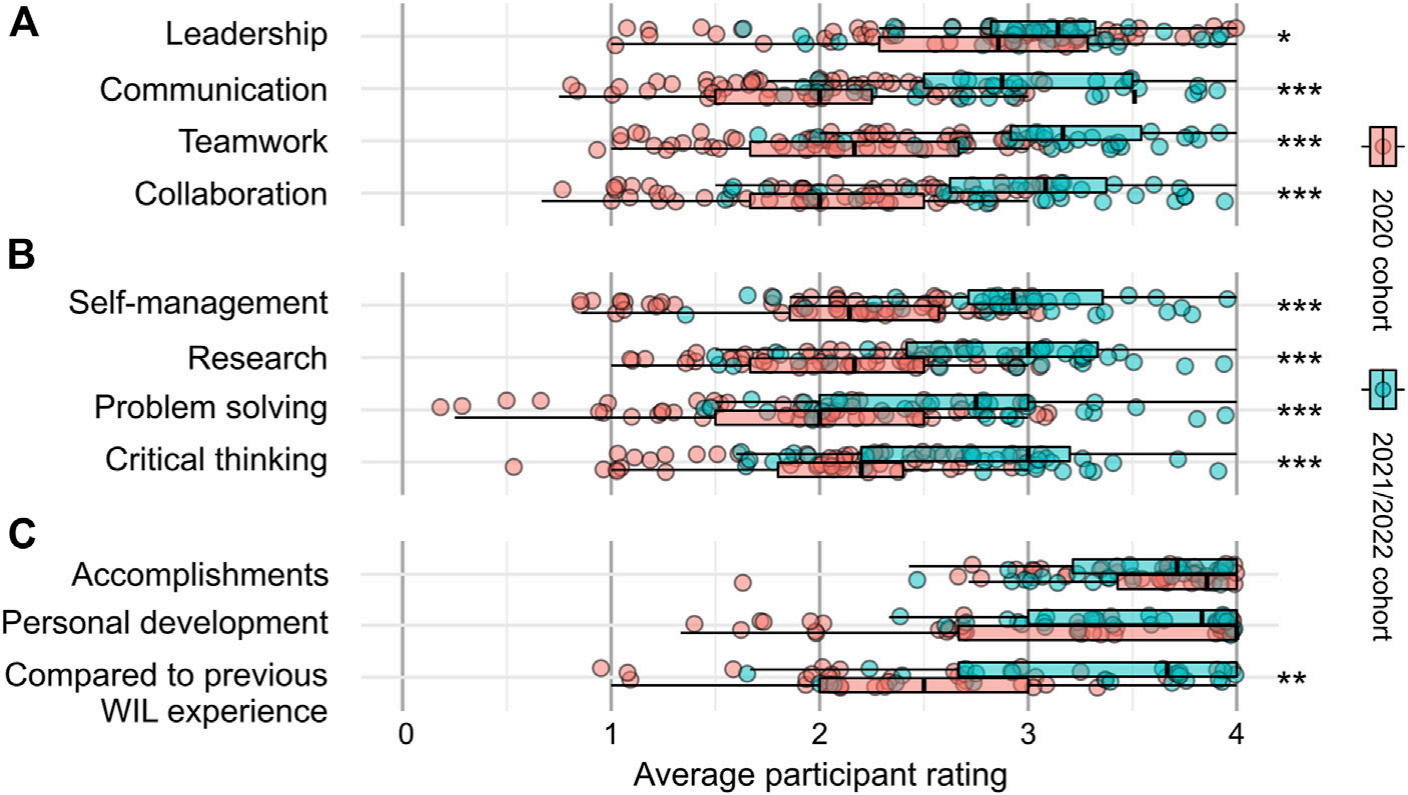
Program improvements. Response distributions grouped by participants from the first iteration of the program (2020 cohort) and participants from later iterations (2021/2022 cohort) grouped by **(A)** interpersonal skills, **(B)** cognitive and intrapersonal skills, and **(C)** personal experiences. Asterisks indicate the upper value of the *p*-value associated with the null hypothesis that the two distributions are not shifted relative to one another (Two-sample Wilcox test, one-sided). One asterisk corresponds to *p* < 0.05, two asterisks correspond to *p* < 0.002, and three asterisks correspond to *p* < 0.0001).

The differences between program iterations were explored further by conducting a statistical comparison of participants’ responses from 2020 to those collected in 2021 and 2022. The results in Figure 5 indicate a significant improvement in interpersonal and intrapersonal skills development. In terms of interpersonal skills (Figure 5A), the median rating increases from roughly two (moderate improvement) to three (significant improvement) in communication, teamwork, and collaboration skills. The same trend is observed for intrapersonal skills (Figure 5B) and certain program aspects (Figure 5C). Notably, more than 50% of 2021 and 2022 respondents reported significant or profound gains in self-management, research, problem-solving and critical thinking skills.

Interestingly, the rating of the BioExperience compared to conventional WIL programs also increased significantly after the first year. This effect is quite striking. The median rating was roughly 2.5 among 2020 respondents and close to 3.8 among 2021 and 2022 respondents. This increase reflects that 76% of respondents (19 of 25) participating in 2021 and 2022 reported that the program is better (24%) or much better (52%) than conventional WIL.

## Discussions and conclusion

The BioExperience Research and Entrepreneurship challenge was created to mitigate the widespread disruption of conventional post-secondary WIL programs caused by the Covid-19 pandemic. The program provides meaningful talent and skills development opportunities through a student-centred, project-based approach inspired by the iGEM competition. The competition has been a unifying force in the synthetic biology community for almost two decades and has allowed countless students to explore biology-based technology and engineering solutions to significant societal problems. We have demonstrated that critical talent and skills development elements of the iGEM competition can be replicated in a program that addresses deficiencies documented in recent research and requires significantly fewer resources than conventional WIL models.

The BioExperience program differs from the iGEM competition by having each team focus on a challenge posed by a program partner, such as a faculty member, a company, or a non-governmental organization. This partner, or team client, supports the team throughout the program in an advisory capacity and is not permitted to assume the role of an employer or supervisor. Students must apply their creativity and ingenuity and use their combined knowledge, skills, and abilities to produce tangible solutions. This independence allows students to develop numerous cognitive, intrapersonal, and interpersonal skills, simultaneously acquiring technical and field-specific competencies that align with their interests, personal needs, and long-term goals.

The responses from BioExperience participants are striking. We knew from experience that the iGEM-inspired approach would provide a learning experience of high quality. Still, we did not anticipate that ∼90% respondents rated the program as excellent or outstanding, or that 19 of 20 respondents reported significant or profound skills improvements in at least one area. Remarkably, three of four respondents found the program to be better or much better than conventional WIL regarding the overall experience, the job-readiness training provided, and the ability to do meaningful work.

Our thematic analysis highlights factors contributing to the learning experience, environment, and outcomes central to the program. The variety of learning opportunities, the relevance to real-world problems, and the impact of personal contributions were highlighted as factors contributing to a high-quality learning experience. More specifically, the independence and self-management afforded by the student-centred learning approach and the program’s collaborative nature were frequently mentioned as superior to conventional WIL.

The interpretation of the survey data results is obscured by the unclear impact of the COVID-19 pandemic on the BioExperience learning environment. For example, guidance and support from advisors were identified as improvement areas but were referenced predominantly by participants in the 2020 program. It is unclear if this change results from advisors being more consistently available in 2021 and 2022 (we recommend at least two hours per week), for example, or students becoming more accustomed to working independently. While many respondents reported that working from home had a negative effect, others highlighted the opportunity to work from home as a benefit of the program. Regardless, our results strongly suggest that the program can provide a high-quality experience without facilities, such as shared office space and classrooms, where participants can work in physical proximity. To improve communication and social cohesion, we now encourage teams to organize regular in-person meetings to use public facilities such as libraries, coffee shops or pubs, and clients to meet occasionally with the team at their workplace.

The analysis also highlights the work products and deliverables, and the development of technical and non-technical skills, as critical factors. Most respondents expressed that they were very satisfied with the project’s tangible outcomes they produced. Although some teams were required to create specific products and deliverables for their client, including physical prototypes, protocols, and educational materials, most were only asked to produce the deliverables required by the program. These deliverables are the same for all teams: a 250-word non-technical project summary and a 2500-word non-technical project description, a two-minute pitch video, a 15-min presentation, and technical reports that are sufficiently detailed for future teams to continue the project. Because of the high satisfaction rate (98% of respondents were satisfied and 60% were very satisfied) and frequent positive references to work products and deliverables, we see no reason to suggest changes to the program.

Cognitive, interpersonal, and interpersonal skills are essential to many employers (BioTalent Canada, 2021) and success in professional and research-based graduate programs (Madan and Teitge, 2013). These skills are difficult to acquire in conventional academic programs, and BioTalent Canada recently called for expanding WIL opportunities (BioTalent Canada, 2020). BioExperience program participants reported that the program improved essential cognitive, interpersonal, and intrapersonal skills. While ∼50% of the respondents reported significant or profound improvement in self-management, problem-solving, critical thinking, collaboration, teamwork, or communication skills, the program appears well-suited for developing research and leadership skills.

Remarkably, 98% of respondents were satisfied with their improvement in non-technical skills, and more than two-thirds were very satisfied. These improvements are also reflected in the roughly 95% of respondents who agree that the program contributed to their personal development by increasing their confidence and enthusiasm for learning, comfort with taking on unfamiliar challenges, and confidence in their ability to investigate and share new ideas. Notably, intrapersonal skills development is vital to emotional wellness, optimism, self-esteem, effective leadership, and educational success (Hindes et al., 2008; Ferreira et al., 2020). It creates the foundation for developing an inquisitive and opportunity-seeking mindset that contributes to success in research-based graduate programs. Nonetheless, because of the importance of non-technical skills development, we recommend further augmenting the student-centred learning model with additional instructor-led learning activities. The goal of these activities is to further support participant skill development by providing them with structured opportunities to learn critical skills, such as creating learning plans and conducting self-reflections, that will make their experience in the BioExperience program more rewarding.

## Data Availability

The definitions used to define higher-order skills used in the study are included in the article/Supplementary Material, further inquiries can be directed to the corresponding author.
